# Implementation science: understanding behaviour change and maintenance

**DOI:** 10.1186/1472-6963-14-S2-O9

**Published:** 2014-07-07

**Authors:** Susan Michie

**Affiliations:** 1 Department of Health Psychology & Centre for Behaviour Change, University College London, UK

## 

Interventions to improve implementation of evidence-based health care have achieved modest and variable success. Improving implementation depends on changing the behaviour of health professionals, managers, commissioners and others working within and with the health care system. Achieving and maintaining behaviour change within the ethical and practical constraints that typically operate remains a formidable challenge. Meeting it requires:

1. a systematic method for analyzing the target behaviours in their context as a starting point for designing an intervention,

2. selecting interventions that are most likely to be effective given this analysis,

3. specifying the intervention in sufficient detail in trial protocols and published reports to allow accurate replication and evidence syntheses, and

4. drawing on relevant theory to guide both the intervention design and evaluation.

This approach can generate evidence about the mechanisms of action of effective interventions and about why interventions vary across different types of behaviour and in different populations and settings. Such evidence is essential for designing more effective interventions. The most effective interventions for both initiating and maintaining behaviour change are those that act simultaneously at many different ‘levels’. A framework for analyzing target behaviours in context and considering the full range of intervention functions and policy categories that may be relevant to the intervention problem is the Behaviour Change Wheel [1,2]. This was derived from a systematic review of 19 published frameworks, none of which were found to contain all the intervention functions know to be relevant. The Behaviour Change Wheel provides a basis for identifying what it would take to achieve the desired behaviour change in terms of changes to Capability, Opportunity and Motivation (the COM-B system). It then links this to 9 intervention functions (Education, Persuasion, Incentivisation, Coercion, Training, Restriction, Environmental Restructuring, Modeling and Enablement) and 7 types of policy that could be used to implement these intervention functions (Mass-media/marketing, Legislation, Fiscal policy, Service provision, Guideline development, Regulation and Environmental/social planning).

Once the intervention strategy has been provisionally established, specific types of behaviour change technique can be selected, guided by evidence, theory and practicalities, to deliver the intervention.
